# Unwell in hospital but not incapable: cross-sectional study on the dissociation of decision-making capacity for treatment and research in in-patients with schizophrenia and related psychoses

**DOI:** 10.1192/bjp.2018.85

**Published:** 2018-08

**Authors:** Benjamin Walter Jack Spencer, Tania Gergel, Matthew Hotopf, Gareth S. Owen

**Affiliations:** 1 Mental Health, Ethics and Law Research Group, Department of Psychological Medicine, Institute of Psychiatry, Psychology and Neuroscience, King's College London and South London and Maudsley National Health Service Foundation Trust, Maudsley Hospital, UK; 2 Mental Health, Ethics and Law Research Group, Department of Psychological Medicine, Institute of Psychiatry, Psychology and Neuroscience, King's College London, UK; 3 Mental Health, Ethics and Law Research Group, Department of Psychological Medicine, Institute of Psychiatry, Psychology and Neuroscience, King's College London and South London and Maudsley National Health Service Foundation Trust, Maudsley Hospital, UK.

## Abstract

**Background:**

Consent to research with decision-making capacity for research (DMC-R) is normally a requirement for study participation. Although the symptoms of schizophrenia and related psychoses are known to affect decision-making capacity for treatment (DMC-T), we know little about their effect on DMC-R.

**Aims:**

We aimed to determine if DMC-R differs from DMC-T in proportion and associated symptoms in an in-patient sample of people with schizophrenia and related psychoses.

**Method:**

Cross-sectional study of psychiatric in-patients admitted for assessment and/or treatment of schizophrenia and related psychoses. We measured DMC-R and DMC-T using ‘expert judgement’ clinical assessment guided by the MacArthur Competence Assessment Tool for Clinical Research, the MacArthur Competence Assessment Tool for Treatment and the legal framework of the Mental Capacity Act (2005), in addition to symptoms of psychosis.

**Results:**

There were 84 participants in the study. Half the participants had DMC-R (51%, 95% CI 40–62%) and a third had DMC-T (31%, 95% CI 21–43%) and this difference was statistically significant (*P* < 0.01). Thought disorder was most associated with lacking DMC-R (odds ratio 5.72, 95% CI 2.01–16.31, *P* = 0.001), whereas lack of insight was most associated with lacking DMC-T (odds ratio 26.34, 95% CI 3.60–192.66, *P* = 0.001). With the exception of improved education status and better DMC-R, there was no effect of sociodemographic variables on either DMC-R or DMC-T.

**Conclusions:**

We have shown that even when severely unwell, people with schizophrenia and related psychoses in in-patient settings commonly retain DMC-R despite lacking DMC-T. Furthermore, different symptoms have different effects on decision-making abilities for different decisions. We should not view in-patient psychiatric settings as a research ‘no-go area’ and, where appropriate, should recruit in these settings.

**Declaration of interest:**

None.

Decision-making capacity (DMC) can be framed as the ability to make a decision that has legal authority when consent is formalised, such as decisions around medical treatment or research. DMC is defined as being specific to the decision in question: different factors will affect the ability to make different decisions, and the presence or absence of DMC for one type of decision does not necessarily imply the status of DMC for other decisions. DMC is a legal concept and the final arbiter or ‘gold standard’ is a decision by the court. However, it is psychopathological symptoms that lead to impairment of DMC and, when finding that DMC is lacking in an individual, these symptoms must be observed to have an impact on DMC. Thus, DMC is a legal construct underpinned by psychopathology.

Schizophrenia and related psychoses (such as schizoaffective disorder, delusional disorder, and acute and transient psychoses) are commonly associated with impairments in DMC.^1^ This may influence the decisions of institutional review boards or research ethics committees when it comes to recruiting research participants with these conditions. However, there is an indisputable need for more research in schizophrenia and related psychoses,^2^ especially with participants who are detained in hospital, severely unwell, or with chronic illness and prominent negative symptoms – people for whom there is evidence of systemic exclusion from research studies.^3^^,^^4^

Participation in human research either requires the consent of participants (with DMC for research, DMC-R), or that of the legally authorised representative in those lacking DMC-R.^5^ If an individual objects to participation, irrespective of their DMC-R, the objection is always respected.^5^ However, the psychopathology of schizophrenia and related psychoses can have a substantial impact on both DMC for treatment (DMC-T) and DMC-R (for a review see Spencer *et al*, 2017^1^). There is a moral imperative to adequately protect people from the consequences of a decision made when DMC is lacking, but also to ensure autonomy is respected and that DMC is maximised. Therefore, to recruit people with schizophrenia and related psychoses to research studies safely, we need to understand the nature of DMC-R when people are acutely unwell.

## Standards of assessing DMC

DMC can be measured as either a binary categorical outcome or on dimensional measures of key abilities. Both the categorical outcome and abilities are defined legally and differ by jurisdiction; although the definitions in the USA and England and Wales are largely viewed to be synonymous.^6^ In England and Wales, these abilities are defined in the Mental Capacity Act (2005) (MCA) as ‘understanding’, ‘retention’, ‘use and weigh’ and ‘communicating a choice’;^7^ whereas in the USA they are defined as ‘understanding’, ‘appreciation’, ‘reasoning’ and ‘expressing a choice’ (the ‘four factors’).^8^ In practice, the routine standard is a clinical or court assessment of DMC returning a binary judgement. This assessment may be framed by legal criteria such as the MCA in the UK or the four-factor model in the USA. We name this standard clinical ‘expert judgement’. Research in the field has predominantly used only dimensional measures of the four factors to facilitate more detailed study of the key abilities. We have previously argued that there are limitations in using these measures alone as they do not necessarily inform DMC, which is inherently binary. For that, one needs to include a measure using an expert judgement standard.^1^

## Current evidence

There is evidence that different psychopathology affects different decisions differently: our systematic review of previous research has shown that insight has the largest effect on DMC-T, whereas neurocognitive deficits have the most impact on DMC-R.^1^ However, no study has ever compared the impact of psychopathology on two different types of decision concomitantly, a necessary feature to control for differing study designs. Few studies to date have investigated DMC-R in an in-patient setting with severely unwell participants, and none of these used an expert judgement measure. We therefore aimed to investigate the proportion and associated symptoms of DMC-R in severely unwell in-patients with schizophrenia and related psychoses compared with DMC-T.

Studies of DMC-R comprise a study assessing DMC-R and a ‘parent study’ (the study that is explained to participants and for which their DMC is assessed). For our comparison of DMC-R and DMC-T, we desired a parent study that was clearly separate from decisions around current treatment to delineate these two decisions. Therefore, we chose a non-therapeutic research project – the ‘National Institute for Health Research (NIHR) BioResource study’ – as we considered that there would be a large overlap between decisions about participation in therapeutic research and decisions about treatment.

## Method

### Participants

We recruited people admitted to eight wards in South London and Maudsley National Health Service Foundation Trust. Eligible participants were approached as soon as possible, from 24 h after admission.

We aimed to study schizophrenia and related psychoses to focus on the core symptoms of ‘schizophreniform’ psychotic illnesses of interest (delusions, hallucinations, thought disorder, negative symptoms, neurocognitive symptoms and lack of insight). With regards to thought disorder, we were particularly interested in those disturbances in thought found in schizophrenia, such as ‘distorted connections between successive thoughts’^9^ and ‘loss of the normal structure of thinking’,^10^ as assessed by the Positive and Negative Syndrome Scale (PANSS)^11^ under item P2 ‘conceptual disorganization’.

Psychiatric diagnosis is fluid. Decisions regarding inclusion and exclusion criteria were made to balance the need to best sample the population with symptoms of schizophreniform psychotic illness, while also obtaining a homogeneous sample to perform the analysis between predictor variables and the outcomes of assessment of DMC-R and DMC-R and to manage possible confounding variables (severe affective symptoms in affective disorders such as depression, or underlying neurological illness such as in organic brain disease). Bipolar affective disorder and related affective psychoses, such as psychotic depression, were excluded because these disorders have separate and distinctive putative mechanisms that influence DMC independently of the symptoms of schizophrenia and related psychoses (see for example Owen, 2013^12^ for a discussion on the mechanisms of affective symptoms that influence DMC-T in severe depression). Despite having prominent affective symptoms by definition, we decided to include schizoaffective disorder as it retained the core symptoms that we wished to investigate. We defined the criteria for the purposes of admission as ‘admission for assessment and/or treatment’ of schizophrenia and related psychoses. This was to exclude those people who were well from a psychosis perspective but were admitted for other reasons, such as social or housing issues.

The inclusion criteria were as follows: age > 18; fluency in English; diagnosis of non-affective psychotic illness as defined by F20–F29^13^ of the ICD-10 (1992) (excluding F21 ‘schizotypal disorder’, which can be considered to be a personality disorder and has only some of the features of schizophrenia^10^) or 295, 297 and 298 of the DSM-IV (1994);^14^ and primary purpose of admission into hospital must have been for the assessment and/or treatment of symptoms of psychosis. The exclusion criteria were current intoxication and previous recruitment into a BioResource study. Participants were offered £10 as compensation for their time. All study interviews were performed by the first author. We were able to recruit participants lacking DMC-R for participation in the present study by seeking the approval of a nominated consultee. In England and Wales, the Mental Health Act (1983) can be used to authorise the detention and treatment of in-patients with mental disorder based on risk. We did not exclude detained people from participating and, in fact, 75% of our participants were formally detained at the time of participation. This study was approved by the Camberwell and St Giles Ethics Committee (reference 15/LO/0427).

### Measures of DMC

We selected the NIHR BioResource as the ‘parent study’ because it is a real non-therapeutic research study that recruits in in-patient settings.^15^ The NIHR BioResource is a biobank study that collects biological (blood and/or saliva) samples and links them to medical data. Key issues of the BioResource include re-contacting participants in the future based on phenotype or genotype, ‘broad consent’ for future research and that participation will have no therapeutic benefit to the participant (*vis-à-vis* the ‘therapeutic misconception’^16^). In liaison with experts in biobanking and our study's Service User and Carer Advisory Group, we distilled the key information about BioResource research that an individual would need to consider to provide valid informed consent. DMC-R was examined by semi-structured interview, using the legal framework for the assessment of capacity under the MCA, dichotomised into a binary outcome.

The interview used the MacArthur Competence Assessment Tool for Clinical Research (MacCAT-CR).^17^ The MacCAT-CR is a semi-structured tool that returns scores on ‘understanding’, ‘appreciation’, ‘reasoning’ and ‘communication’. We adapted the tool for BioResource research and, using similar methods as described by Cairns 2005^18^ and Kim 2011,^19^ were able to show an excellent inter-rater reliability with a pairwise kappa of 0.8 (corresponding author for details). We measured DMC-T using a similar approach as for DMC-R. Here, the decision was framed around ‘admission and treatment’ in hospital (seen conjunctively) and was informed by relevant information about the participant's diagnosis, symptoms, purpose and reasons for admission, and recommended treatment from the case notes and discussion with the clinical team. We structured the interview using the MacArthur Competence Assessment Tool for Treatment (MacCAT-T),^20^ which is structured similarly to the MacCAT-CR.

### Other assessments

Basic demographics were collected from participants, clinical diagnosis under ICD-10^13^ was obtained from the assigned diagnosis in the case notes and validated using the Operational Criteria Checklist.^21^ Participants underwent assessments using the PANSS^11^ (this includes a measure of insight, PANSS item G12), Young's Mania Rating Scale (YMRS),^22^ the 17-item Hamilton Rating Scale for Depression (HRSD-17),^23^ Health of the Nation Outcome Scales (HoNOS)^24^ and the Clinical Global Impression – Severity Scale (CGI-S).^25^

We developed a Neurocognitive Assessment Battery for the study that included: verbal and category fluency,^26^ Digit Span,^27^ Digit Symbol Substitution Test,^27^ Letter-Number Sequencing,^27^ story memory test^28^ and Trail Making Test parts A and B.^29^ These tests were selected for brevity of assessment and to focus on deficits in verbal and working memory present in those with schizophrenia and related psychoses.^30^^,^^31^

### Statistical analysis

All analyses were performed using Stata version 14 (StataCorp, Texas). Measures of symptoms were converted into *z*-scores based on the sample mean and standard distribution to facilitate direct comparison. Where the distribution of scores was skewed, the score was trichotomised into high, medium or low, based on the sample range. Within the neurocognitive measures, the number of missing items was very high (>70% of cases were missing at least one item). This was a severely unwell sample and missing data were commonly due to participant disengagement from further assessments. We were interested in the core symptoms of psychosis, such as hallucinations and delusions. Therefore, we did not impute these missing data and we performed a ‘complete case’ analysis, selecting the relevant measures from the Neurocognitive Assessment Battery with the most data (Digit Span for working memory; Logical Memory 1 for short-term memory). To enable direct comparison between the PANSS and neurocognitive items, we restricted the analysis of these items to those cases with data for all measures (the ‘restricted data set’).

## Results

### Study sample

A total of 84 participants completed the DMC-R assessments. Consultee approval to consent of the study was obtained in three participants, with one subsequently regaining DMC-R for the present study. Basic sociodemographics and measures on the symptom scores of the sample are provided in Table 1.

**Table 1:** Descriptive sociodemographics and symptom scales of participants

Sociodemographics	
Age (*n* = 84)	38.40 (12.21)
Gender (*n* = 84)	
Number female	21 (25%)
Ethnicity (*n* = 80)	
White British	21 (25%)
Black African	21 (25%)
Black Caribbean	17 (20%)
Mixed	12 (14%)
Other Black and minority ethnic	5 (6%)
White other	4 (5%)
Education (*n* = 84)	
GCSE/10th grade or below	37 (44%)
A-Level/12th grade or above	47 (56%)
Current employment (*n* = 84)	
Employed	11 (13%)
Unemployed	73 (87%)
Previous involvement in research (*n* = 84)	
Other research – prior involvement	38 (45%)
No prior research discussions	46 (55%)
Days from admission to recruitment (*n* = 84)	11 (17)^a^
Clinical variables	
** **Primary diagnosis (*n* = 84)	
** **F20: schizophrenia	61 (73%)
** **F25: schizoaffective disorder	14 (17%)
** **F22: persistent delusional disorder	4 (5%)
** **Other (F23, F28, F29)	5 (5%)
** **Detained at time of interview (*n* = 84)	
** **Informal	21 (25%)
** **Detained	63 (75%)
** **PANSS scores	
** **Total score (*n* = 64)	68.66 (15.33)
** **Positive symptom score (*n* = 78)	20.77 (6.47)
** **Negative symptom score (*n* = 78)	12 (12)^a^
** **General symptom score (*n* = 69)	33.13 (6.88)
Insight	
** **Item G12 insight (*n* = 77)	4.44 (1.63)
** **Global illness severity	
** **CGI (*n* = 84)	4.08 (0.88)
** **HoNOS (*n* = 81)	12.70 (5.89)
** **Affective symptoms	
** **YMRS (*n* = 65)	12.06 (7.27)
** **HRSD-17 (*n* = 65)	5 (6)^a^
** **Neurocognition	
** **Neurocognitive *z*-score (*n* = 25)	−1.28 (0.98)

Results are presented as means and s.d. unless specified. PANSS, Positive and Negative Syndrome Scale; CGI, Clinical Global Impression; HoNOS, Health of the Nation Outcome Scales; YMRS, Young's Mania Rating Scale; HRSD-17, 17-item Hamilton Rating Scale for Depression.

a. Median and interquartile range.

### Measures of DMC-R and DMC-T

We found that half of the participants had DMC-R (51%, 95% CI 40–62%), compared with a third who had DMC-T (31%, 95% CI 21–43%). This difference was highly statistically significant: *P* < 0.01. Table 2 shows the distribution of people having DMC-R *v.* DMC-T. Although in most cases participants either lacked or had both DMC-R and DMC-T (*n* = 59, 74%), there were dissociations: 23% (*n* = 18) had DMC-R but lacked DMC-T, and 4% (*n* = 3) lacked DMC-R but had DMC-T.

**Table 2:** Presence of DMC-R *v.* DMC-T

		DMC-T	
		Present	Absent
DMC-R	Present	22 (28%)	18 (23%)
	Absent	3 (4%)	37 (46%)

DMC-R, decision-making capacity for research; DMC-T, decision-making capacity for treatment.

### Associations with DMC-R and DMC-T

#### Sociodemographics

Associations between sociodemographic variables and DMC-R and DMC-T are presented in Table 3. There were no associations with age, gender, ethnicity, previous involvement in research and current employment with either DMC-R or DMC-T. Highest educational attainment (A-Level/12th grade or above) was associated with having DMC-R (odds ratio 0.31, 95% CI 0.12–0.75, *P* = 0.010); however there were no associations with highest educational attainment and DMC-T.

**Table 3:** Odds ratios of sociodemographic predictor variables on lacking DMC-R and DMC-T

(*n* DMC-R, *n* DMC-T)	DMC-R			DMC-T		
	Odds ratio	95% CI	*P*	Odds ratio	95% CI	*P*
Age (*n* = 84, 80)						
Years	1.03	0.99–1.07	0.116	1.04	0.99–1.08	0.100
Gender (*n* = 84, 80)						
Male	1			1		
Female	0.56	0.20–1.54	0.260	0.80	0.27–2.33	0.676
Ethnicity (*n* = 80, 76)						
White British	1			1		
Black African	0.56	0.17–1.91	0.356	0.64	0.17–2.38	0.508
Black Caribbean	0.67	0.18–2.41	0.537	0.94	0.23–3.92	0.936
Mixed	0.54	0.13–2.25	0.394	1.14	0.22–5.87	0.873
Other Black and minority ethnic	1.13	0.15–8.21	0.908	1.71	0.16–18.73	0.659
White other	2.25	0.20–25.37	0.512	^a^		
Involvement in research (*n* = 84, 80)						
No prior involvement in research	1			1		
Previous involvement in research	1.09	0.46–2.58	0.843	1.14	0.44–2.95	0.786
Education (*n* = 84, 80)						
GCSE/10th grade or below	1			1		
A-Level/12th grade or above	**0**.**31**	**0.12–0.75**	**0**.**010**	0.63	0.24–1.66	0.348
Current employment (*n* = 84, 80)						
Unemployed	1					
Employed	0.86	0.24–3.06	0.811	2.25	0.45–11.28	0.324
Diagnosis (*n* = 84, 80)						
Schizophrenia	1			1		
Schizoaffective disorder	0.85	0.27–2.71	0.782	0.61	0.17–2.20	0.454
Delusional disorder	^b^			^b^		
Other	0.21	0.02–2.01	0.176	0.66	0.10–4.29	0.662
Detained at time of interview (*n* = 84, 80)						
Informal	1			1		
Detained	1.79	0.65–4.91	0.260	**3**.**41**	**1.16–9.96**	**0**.**025**
Days until recruited (*n* = 84)						
Days until recruited	1.01	0.99–1.03	0.282	1.00	0.98–1.02	0.832

Significant values have been highlighted in bold type.

DMC-R, decision-making capacity for research; DMC-T, decision-making capacity for treatment.

a. All participants of White other ethnicity lacked DMC-T.

b. All participants with delusional disorder (*n* = 4) had DMC-R and lacked DMC-T.

#### Clinical factors and symptoms

Associations between clinical factors and symptoms and DMC-R and DMC-T are presented in Table 4. Diagnostic subtype was not associated with lacking either DMC-R or DMC-T, with the exception of delusional disorder, in which all participants had DMC-R but lacked DMC-T (*n* = 4). Detention in hospital was associated with lacking DMC-T (odds ratio 3.41, 95% CI 1.16–9.96, *P* = 0.025) but not DMC-R.

**Table 4:** Odds ratios of symptom predictor variables on lacking DMC-R and DMC-T

(*n* DMC-R, *n* DMC-T)	DMC-R			DMC-T		
	Odds ratio	95% CI	*P*	Odds ratio	95% CI	*P*
Global Severity Assessments						
CGI score (*n* = 84, 80)	**6**.**59**	**2.93–14.81**	**<0**.**001**	**3**.**73**	**1.88–7.40**	**<0**.**001**
HoNOS (*n* = 81, 77)	**2**.**50**	**1.48–4.23**	**0**.**001**	**1.82**	**1.07–3.09**	**0.026**
Affective symptoms						
HRSD-17 T score^a^ (*n* = 65, 65)	0.31	0.07–1.35	0.119	0.48	0.16–1.48	0.203
YMRS score (*n* = 65, 65)	**3**.**00**	**1.53–5.88**	**0**.**001**	**3**.**74**	**1.69–8.31**	**0**.**001**
Psychosis and neurocognitive symptom measures – restricted data set *n* = 46						
PANSS total symptom	**8**.**99**	**2.60–31.13**	**0**.**001**	**2**.**36**	**1.08–5.15**	**0**.**031**
PANSS positive symptom	**4**.**00**	**1.70–9.41**	**0**.**002**	**3**.**88**	**1.44–10.42**	**0**.**007**
PANSS negative symptom T score^a^	2.33	0.91–5.98	0.079	0.89	0.36–2.22	0.805
Delusions	**2**.**13**	**1.09–4.16**	**0**.**026**	**3**.**67**	**1.62–8.32**	**0**.**002**
Thought disorder	**5**.**72**	**2.01–16.31**	**0**.**001**	1.82	0.85–3.90	0.125
Hallucinations T score^a^	1.41	0.55–3.60	0.473	1.47	0.52–4.13	0.466
Insight	1.86	0.91–3.79	0.089	**26**.**34**	**3.60–192.66**	**0**.**001**
Digit Span	1.67	0.76–3.67	0.202	1.94	0.89–4.27	0.098
Logical Memory 1 recall	**2**.**68**	**1.43–5.02**	**0**.**002**	1.38	0.81–2.34	0.232

All symptom measures have been converted into *z*-scores based on the sample mean and s.d. to facilitate direct comparison unless indicated.

Significant values have been highlighted in bold type.

DMC-R, decision-making capacity for research; DMC-T, decision-making capacity for treatment; CGI, Clinical Global Impression; HoNOS, Health of the Nation Outcome Scales; HRSD-17, 17-item Hamilton Rating Scale for Depression; YMRS, Young's Mania Rating Scale; PANSS, Positive and Negative Syndrome Scale.

a. T score: trichotomised by sample range into scores of low 1, medium 2 or high 3 due to skewed distribution.

Measures of total psychotic symptom burden or overall illness severity (PANSS total score, CGI and HoNOS) were associated with worse DMC-R and DMC-T. There were no associations with HRSD-17 and lacking either DMC-R or DMC-T. Manic symptoms were associated with lacking both DMC-R and DMC-T with similar effect sizes (odds ratios 3.00, 3.74).

The restricted data set did not differ significantly from the full data set on age (mean 39.93 (12.56) *v.* 38.40 (12.21)), gender (female *n* = 13, 28% *v.* 21, 25%) and education A-Level/12th grade and above (*n* = 30, 65% *v.* 47, 56%). With the restricted data set, PANSS positive symptoms were associated with both lacking DMC-R and DMC-T (odds ratios 4.00, 3.88); although PANSS negative symptoms were not associated with lacking either DMC-R or DMC-T, significance was just missed for DMC-R (odds ratio 2.33, 95% CI 0.91–5.98, *P* = 0.079). In terms of the individual key symptoms of psychosis, hallucinations were not associated with lacking either DMC-R or DMC-T, delusions were associated with lacking both DMC-R and DMC-T (odds ratios 2.13, 3.67) but thought disorder was only associated with lacking DMC-R (odds ratio 5.72, 95% CI 2.01–16.31, *P* = 0.001). Worse Digit Span performance was not associated with lacking either DMC-R or DMC-T, but worse Logical Memory 1 performance was associated with lacking DMC-R (odds ratio 2.68, 95% CI 1.43–5.02, *P* = 0.002). Lack of insight had the largest effect on lacking DMC-T (odds ratio 26.34, 95% CI 3.60–192.66, *P* = 0.001), but narrowly missed significance with lacking DMC-R (odds ratio 1.86, 95% CI 0.91–3.79, *P* = 0.089). When the data set was unrestricted, thought disorder was associated with lacking DMC-T (odds ratio 2.12, 95% CI 1.22–3.68, *P* = 0.008) and lack of insight was associated with lacking DMC-R (odds ratio 2.76, 95% CI 1.55–4.90, *P* = 0.001).

## Discussion

### Main findings

We have shown that DMC-R is different from DMC-T in terms of the proportion of people in which is it present while unwell in hospital and the associated symptoms with lacking DMC. When unwell, around half of the participants with schizophrenia and related psychoses had DMC-R, which is more than those with DMC-T. Symptoms that had the largest effect on DMC-R were related to disorganised thinking and poor short-term memory (thought disorder and Logical Memory 1). In contrast, the largest and most significant effect on DMC-T was lack of insight.

Consistent with other work, we did not find an association with sociodemographic variables and either DMC-R and DMC-T, with the exception of an effect of greater years of education and having DMC-R. Out of the core symptoms of psychosis, hallucinations and delusions, only delusions had an effect on DMC-R and DMC-T, whereas hallucinations had no effect. In all measures of overall psychosis symptom severity used in this study, higher symptoms scores were associated with worse DMC-R and DMC-T.

#### Insight

The finding that insight has a central role in DMC-T is consistent with previous work.^1^ The association with DMC-R just missed significance in the restricted data set, but was significantly associated in the unrestricted data set. We deliberately selected a parent study which we believed would not require insight into one's own illness. One explanation for this could be that lack of insight is associated with a reduction of meta-cognitive ability^32^ and thus would have an impact on decision-making in general. However, it is possible that there remains a role for insight in decision-making, even in decisions regarding non-therapeutic research.

#### Thought disorder and understanding

The participants that lacked DMC-R but had DMC-T bear special consideration. In two out of three of these participants, the main reason for lacking DMC-R was a lack of understanding due to thought disorder. But this was not severe enough to affect understanding in DMC-T and participants were protected from the effects of the thought disorder due to prior knowledge. DMC-R required the ability to understand and process novel information, but this was not necessarily the case in DMC-T and this may explain the strong effect of short-term memory performance and thought disorder on DMC-R rather than DMC-T. There are many studies regarding the utility of educational interventions to support understanding for research participation in schizophrenia and related psychoses (see for example^33^^,^^34^). Our findings both support this and the importance of decisional support tools around understanding in research participation discussions.

#### Decision and person specificity of DMC-R and DMC-T

Our data strongly support the legal and conceptual premise of decision specificity. We have found that two different decisions differ in both the proportion of people having DMC and the associated symptoms. Of interest is the impact of the cognitive symptoms such as short-term memory and thought disorder (although a primary symptom of psychosis thought disorder clearly has a direct cognitive effect) and their strong impact on DMC-R but limited impact on DMC-T. One might assume that these would affect all decisions equally, however we found evidence to the contrary. Although it is self-evident that some decisions may require more detailed understanding than others, the impact of symptoms on these decisions may be different, especially if there is prior knowledge of the subject area. Therefore, when interpreting our results, it is worth reflecting that the impact of symptoms will not just vary by decision, but also by individual and their previous life experience and knowledge.

### Limitations

#### Power and missing data

We were powered to detect a large effect size between the symptom measures and DMC-R and DMC-T, which we deemed to be clinically relevant. Because this is a severely unwell sample, our study is inevitably limited by missing data, particularly within the neurocognitive assessment. To compensate for this, we restricted the analysis of PANSS and neurocognitive items for cases in which we had full data, so that direct comparisons of symptoms and associations with DMC-R and DMC-T could be made. This limits the power of the study. The results do not substantially differ from the unrestricted data set analysis other than for those already mentioned and they enable direct comparison, which is a limitation of the unrestricted data set.

#### Selection bias

Our primary goal was to explore the proportion of DMC-R in the in-patient population and we designed our study accordingly to minimise the risk of selection bias. However, bias likely remained: The level of PANSS negative symptoms was very low in our sample (total score of 12 out of a minimum and maximum range of 7–49). This is unsurprising considering the substantial motivation required to participate in a research study comprising multiple interviews taking >90 min in total. A high proportion of people in our study reported previous participation in research. This may indicate that people who were interested and open to participate in research in general may also be likely to wish to participate in a research study. Finally, it was not possible for the research team to approach many individuals due to risk of violence or sexual disinhibition. We consider these selection biases to be of limited concern because, with consent to research, we are concerned with the population that will safely volunteer to participate in research when severely unwell in hospital; these biases would not have significantly affected recruitment from that population.

### Summary

To our knowledge this is the first study to investigate DMC-R in an in-patient setting with severely unwell participants, using a categorical outcome of the expert judgement standard. This is also the first study to directly compare two different DMC decisions for the same individual as well as their symptom and sociodemographic associations, thereby exploring the decision specificity of DMC and demonstrating its presence and impact.

We have shown that, even when severely unwell, people with schizophrenia and related psychoses commonly retain DMC-R for non-therapeutic research, even when lacking DMC-T. Furthermore, different symptoms have different effects on decision-making abilities for different decisions. Therefore, we should not make assumptions about an individual's DMC-R based on their DMC-T, their degree of illness or their symptom profile. To do so risks the continued exclusion of a patient group that we know has high rates of DMC-R and for whom research is urgently needed to improve care. We should not view in-patient psychiatric settings as a research no-go area and, with suitable safeguards, our results suggest we should recruit in these settings. Just because someone is in hospital with schizophrenia or related psychoses does not mean they are incapable. If we talk to them about research projects they may want to – and be able to – participate.
